# Targeting COX-2/PGE_2_ Pathway in HIPK2 Knockdown Cancer Cells: Impact on Dendritic Cell Maturation

**DOI:** 10.1371/journal.pone.0048342

**Published:** 2012-11-07

**Authors:** Alessia Garufi, Giuseppa Pistritto, Claudia Ceci, Livia Di Renzo, Roberta Santarelli, Alberto Faggioni, Mara Cirone, Gabriella D’Orazi

**Affiliations:** 1 Department of Experimental Oncology, Molecular Oncogenesis Laboratory, Regina Elena National Cancer Institute, Rome, Italy; 2 Department of Neuroscience, Section of Pharmacology, University “Tor Vergata”, Rome, Italy; 3 Department of Experimental Medicine, Institute Pasteur-Foundation Cenci Bolognetti, “Sapienza” University, Rome, Italy; 4 Department of Medical, Oral and Biotechnological Sciences, University “G. d’Annunzio”, Chieti, Italy; University of Oslo, Norway

## Abstract

**Background:**

Homeodomain-interacting protein kinase 2 (HIPK2) is a multifunctional protein that exploits its kinase activity to modulate key molecular pathways in cancer to restrain tumor growth and induce response to therapies. For instance, HIPK2 knockdown induces upregulation of oncogenic hypoxia-inducible factor-1 (HIF-1) activity leading to a constitutive hypoxic and angiogenic phenotype with increased tumor growth *in vivo*. HIPK2 inhibition, therefore, releases pathways leading to production of pro-inflammatory molecules such as vascular endothelial growth factor (VEGF) or prostaglandin E2 (PGE_2_). Tumor-produced inflammatory mediators other than promote tumour growth and vascular development may permit evasion of anti-tumour immune responses. Thus, dendritic cells (DCs) dysfunction induced by tumor-produced molecules, may allow tumor cells to escape immunosurveillance. Here we evaluated the molecular mechanism of PGE_2_ production after HIPK2 depletion and how to modulate it.

**Methodology/Principal findings:**

We show that HIPK2 knockdown in colon cancer cells resulted in cyclooxygenase-2 (COX-2) upregulation and COX-2-derived PGE_2_ generation. At molecular level, COX-2 upregulation depended on HIF-1 activity. We previously reported that zinc treatment inhibits HIF-1 activity. Here, zinc supplementation to HIPK2 depleted cells inhibited HIF-1-induced COX-2 expression and PGE_2_/VEGF production. At translational level, while conditioned media of both siRNA control and HIPK2 depleted cells inhibited DCs maturation, conditioned media of only zinc-treated HIPK2 depleted cells efficiently restored DCs maturation, seen as the expression of co-stimulatory molecules CD80 and CD86, cytokine IL-10 release, and STAT3 phosphorylation.

**Conclusion/Significance:**

These findings show that: 1) HIPK2 knockdown induced COX-2 upregulation, mostly depending on HIF-1 activity; 2) zinc treatment downregulated HIF-1-induced COX-2 and inhibited PGE_2_/VEGF production; and 3) zinc treatment of HIPK2 depleted cells restored DCs maturation.

## Introduction

Homeodomain interacting protein kinase 2 (HIPK2) is a multifunctional kinase whose activity restrains tumor progression. HIPK2 phosphorylates and activates oncosuppressor p53 for apoptotic function in response to drugs [1], [2]. HIPK2 also represses signaling pathways involved in tumor progression, such as Wnt/β-catenin, by means of its catalytic activity and transcription repression function [3], [4]. More recently, we found that HIPK2 inhibits the hypoxia-inducible factor 1α (HIF-1α) subunit at transcriptional level. HIF-1 is an heterodimeric transcription factor that induces the transcription of more than 60 genes involved in angiogenesis, glucose metabolism and invasion [5]. HIF-1 consists of the HIF-1β subunit, constitutively expressed in cells, and the oxygen-sensitive HIF-1α subunit. Increased HIF-1α levels are frequently found in many human cancers and are stimulated by low oxygen but also by genetic alterations. In this regard, HIPK2 knockdown induces HIF-1α upregulation with increased HIF-1 activity leading to vascular endothelial growth (VEGF) production, tumor angiogenesis and chemoresistance [6], [7]. In an attempt to target HIF-1 activity, we previously demonstrated that zinc ions allow HIF-1α protein degradation in cancer cells leading to repression of HIF-1 pathway *in vitro* and *in vivo* [8] with restoration of chemosensitivity [9].

Other than inducing VEGF expression, HIPK2 knockdown leads to increased prostaglandin E2 (PGE_2_) biosynthesis that correlates with tumor growth *in vivo* [10]. PGE_2_ is the most abundant prostaglandin found in colorectal cancer and favours tumor growth by stimulating proliferation, angiogenesis, and invasiveness, and by inhibiting apoptosis [11]. PGE_2_ activates components of the oncogenic Wnt signaling system leading to stabilization and activation of β-catenin as transcription factor to induce the expression of several genes, including cyclooxygenase-2 (COX-2), c-myc, cyclin D1, and PPARδ that are involved in tumor progression [12].

The generation of prostaglandins via COX-2 is an important pathway in the pathogenesis of colorectal cancer but also of other cancers [13]. COX-2, an inducible form of cyclooxygenase, catalyses the conversion of arachidonic acid (AA) to endoperoxide intermediates that are ultimately converted to prostaglandins (PGE2, PGD2, PGF2α, PGI2, and TxA2). COX-2 is highly induced in response to inflammatory mediators, growth factors, oncogene activation, and tumor promoters [14]. Therefore, elevated levels of COX-2 have been found in many cancer types [15]–[17]. Transcriptional control of the COX-2 gene depends on the molecular machinery interacting with the COX-2 promoter, which seems to be controlled through the activity of various signaling pathways [18]. Among them, oncogenic pathways such as the Wnt/β-catenin signaling [19] or HIF-1 [20] may enhance COX-2 gene transcription. It has been found that HIF-1-activated COX-2/PGE_2_ axis induces VEGF and promotes angiogenesis [21] which in turn potentiates HIF-1 transcriptional activity [22]. Such crosstalk among signaling pathways leads to an autoregulatory loop which is beneficial for tumor progression. Therefore, targeting HIF-1 is a functional strategy to abolish pathways involved in tumor progression, such as COX-2/PGE_2_/VEGF.

Tumor cells often produce inflammatory molecules that in the microenvironment, other than promoting tumour growth and vascular development, enable evasion of anti-tumour immune responses [23]. PGE_2_, as other tumor released factors, has been shown to suppress the immune response and allow tumor cells to escape immunosurveillance by inducing, for instance, local and systemic dendritic cells (DCs) dysfunction [24], [25]. DCs are powerful antigen-presenting cells (APCs) and as such have a crucial role in initiating an immune-response. DCs are highly specialized in antigen capture, processing and presentation, and after maturation they express co-stimulatory molecules (i.e., CD80 and CD86) which active T lymphocytes [26]. Their role in eliciting tumor-specific responses and subsequent tumor regression has been extensively demonstrated through *in vivo* vaccine stimulation or *ex vivo* DC immunotherapy [26]. Impairment of DCs maturation may depend by tumor-released factors thereby inducing immunosuppression. Thus, a defect in the DC system is one of the main factors responsible for tumor escape [27], [28].

On the basis of the above observations, the aim of this study was first to evaluate the role of COX-2 in PGE_2_ generation following HIPK2 depletion. We found that HIPK2 knockdown led to HIF-1-induced COX-2 upregulation and COX-2-derived PGE_2_ production. Interestingly, zinc treatment downregulated COX-2 expression and inhibited PGE_2_ generation and its signaling pathways, as well as HIF-1-induced VEGF. Then, at functional level, while conditioned media of both siRNA control and HIPK2 depleted cells inhibited DCs maturation, only conditioned media of zinc-treated HIPK2 depleted cells, which showed strong PGE_2_ and VEGF downregulation, efficiently restored DCs maturation.

## Materials and Methods

### Ethics Statement

The study was approved by the ethical Committee of Policlinico Umberto I, Sapienza University, Rome, Italy.

### Cells, Culture Condition, Treatments, and Conditioned Media

Human RKO (colon cancer) and the stably HIPK2-interfered RKO-siHIPK2 [29] cells were routinely maintained in RPMI-1640 (Life-Technology-Invitrogen) medium, while HCT116 (colon cancer), 293 (human embryonic renal cells) and the Doxyclyclin (Dox)-inducible MCF7 (breast cancer) (MCF7indsi/HIPK2) cells expressing HIPK2-interference [30], were routinely maintained in DMEM (Life-Technology-Invitrogen) medium, all containing 10% heat-inactivated fetal bovine serum (FBS), 100 units/mL penicillin/streptomycin, and glutamine, in 5% CO_2_ humidified incubator at 37°C. For zinc supplementation, subconfluent cells were treated with 100 µM ZnCl_2_ for 24 h. For inducible HIPK2 knockdown, Dox (1 µg/mL) was added to MCF7indsi/HIPK2 cells every 3 days until HIPK2 knockdown was successfully reached (usually in about 5 days). After HIPK2 knockdown was reached, cells were cultured without Dox for additional 5 days for reversion of HIPK2 depletion.

To obtain the conditioned medium (CM), RKO siRNA control and siHIPK2 depleted cells were seeded at 6×10^5^/60 mm2 Petri dish and cultivated until 60% confluence. Thereafter, the medium was replaced and the supernatants (that is, conditioned media) were harvested 48 h later. ZnCl_2_ (100 mM) was added for 24 h.

### RNA Extraction and Reverse Transcription (RT)-PCR Analysis

Cells were harvested in TRIzol Reagent (Invitrogen) and total RNA was isolated following the manufacturer’s instruction. cDNA was syntesized from 2 µg of total RNA with MuLV reverse transcriptase kit (Applied Biosystems). Semi-quantitative RT-PCR was carried out by using Hot-Master Taq polymerase (Eppendorf) with 2 µl cDNA reaction and genes specific oligonucleotides under conditions of linear amplification. PCR was performed in duplicate in two different sets of cDNA. PCR products were run on a 2% agarose gel and visualized by ethidium bromide staining using UV light. The housekeeping β-actin or 28S genes were used as internal standard. Densitometric analysis was applied to quantify specific mRNA levels compared to internal standard. Data presented are representative of at least three independent experiments.

### Western Immunoblotting

Total cell extracts were prepared by incubating at 4°C for 30 min in lysis buffer (50 mmol/L Tris-HCl, pH 7.5, 150 mmol/L NaCl, 150 mmol/L KCl, 1 mmol/L dithiothreitol, 5 mmol/L EDTA, pH 8.0, 1% Nonidet P-40) plus a mix of protease inhibitors (Sigma Chemical Company) and phosphatase inhibitors, and resolved by SDS-polyacrylamide gel electrophoresis. Proteins were transferred to a polyvinylidene difluoride (PVDF) membrane (Millipore). Membranes were blocked with 5% nonfat dry milk in PBS and incubated with primary antibodies that recognize COX-2 (Cayman Chemical), β-catenin (Santa Cruz Biotechnology), cyclin D1 (M-20, Santa Cruz, kindly provided by Marco Crescenzi, ISS, Rome, Italy), mouse monoclonal anti-HIF-1α (Novus Biologicals, UCS Diagnostic, Italy), p-STAT3 (Y705), total STAT3 (both from Cell Signaling Technology), and β-actin (Calbiochem). Secondary antibody conjugated to horseradish peroxidise (Bio-Rad) was used at 1∶5000. Immunoreactivity was detected by enhanced chemiluminescence kit (ECL kit, Amersham Corporation).

### Transfection and Plasmids

293 cells were transfected by using the N,N-bis-(2-hydroxyethyl)-2amino-ethanesulphonic acid-buffered salinr (BBS) version of the calcium phosphate procedure [31], while RKO and HCT116 were transfected by using the cationic polymer LipofectaminePlus method (Invitrogen), according to the manufacture’s instruction. The amount of plasmid DNA was equalized in each sample by supplementing with empty vector and transfection efficiency was visualized with the use of a co-transfected GFP expression vector. The expression plasmids used were: the dominant negative form of HIF-1α without DNA binding domain and transactivation domain (pCEP4-HIF-1α-DN) [32] (kindly provided by B.H. Jiang, Nanjing Medical University, China), HIF-1α expression vector (kindly provided by A. Farsetti, National Research Council, Rome, Italy) and HA-VHL expression vector (kindly provided by WK Rathmell, University of North Caroline at Chapel Hill, USA).

### RNA Interference

Cells were plated at semiconfluence in 35 mm dishes the day before transfection. Control-siRNA and specific siHIF-1α (Dharmacon), or pSUPER-HIPK2 and control pSuper vectors [29] were transfected overnight using LipofectaminePlus reagent (Invitrogen). 36 h later cells were treated with 100 µM ZnCl_2_ for 24 h. Transfection efficiency was visualized with the use of a co-transfected GFP expression vector under a fluorescent microscope. The efficiency of knockdown was confirmed by RT-PCR analysis.

### ELISA Assay

Cells grown to subconfluence in 60 mm Petri dishes were cultured for 24 h with 0.5% FBS before adding fresh medium with 10% FEB with or without COX-2 selective inhibitor NS-398 (Cayman Chemical, Ann Arbor, MI, USA) at 50 nM for 24 h or 100 µM ZnCl_2_ for 24 h with. PGE_2_ and VEGF detection in the cell-conditioned media were determined in triplicate by enzyme-linked immunosorbent assay (ELISA) using, respectively, the Prostaglandin E2 EIA kit (Cayman Chemical) and the Quantikine human VEGF immunoassay kit (R&D Systems, Minneapolis, MN), according to manufacturer’s instructions. PGE_2_ and VEGF levels were normalized to cell number and expressed as pg/10^6^ cells/ml.

For IL-10 production by DCs, conditioned media from siRNA control and siHIPK2 depleted cells, untreated or treated with 100 µM ZnCl_2_ for 24 h, were added to DC culture in 96 well plates, 1/1 (vol/vol) with medium containing IL-4 and GM-CSF, for 24 h. Afterword, TNF-α was added for 48 h for DCs maturation. IL-10 production by DCs was then assayed by ELISA using commercially available reagents and standards (RayBiotech, Inc.). The supernatants were added in duplicate to appropriate pre-coated plates. After the plates were washed, horseradish peroxidase-conjugated detection antibody was added. The substrate used for color development was tetramethylbenzidine (TMB). The optical density was measured at 450 nm with a microplate reader (Multiskan Ex, Thermo Labsystem). The minimum detection dose of IL-10 is typically less than 1 pg/ml.

### Generation of Monocyte-derived Dendritic Cells (DCs) and DCs Maturation

To generate monocyte-derived DCs, human peripheral blood mononuclear cells (PBMC), obtained from healthy donors (under informed consent) were isolated by Fycoll-Paque gradient centrifugation (Pharmacia, Uppsala, Sweden) from buffy coats. CD14+ monocytes were positively selected using anti-CD14 MAb-conjugated magnetic microbeads (Miltenyi Biotec, Auburn, California, USA). Purified monocytes were cultured at a density of 10^6^ cells/3 ml in 12-well plates for 6 days in RPMI 1640 (Euroclone) containing 10% fetal calf serum (FCS), 2 mM L-glutamine, 100 U/ml penicillin G, 100 mg/ml streptomycin and recombinant human granulocyte-macrophage colony stimulating factor (GM-CSF, 50 ng/ml) and interleukin 4 (IL-4, 20 ng/ml) (Miltenyi Biotec) to generate the immature DC (iDC). Cytokines were replenished every other day along with 20% fresh medium. For iDC activation, human recombinant TNF-α (20 ng/mL; Miltenyi Biotec) was added at day 6 for 48 h to induce DCs maturation.

### Analysis of DC Phenotype by Flow Cytometry

Purified monocytes were cultured for 6 days with GM-CSF and IL-4, with or without 20% (vol/vol) of conditioned media (CM) from cancer cells untreated or treated with 100 µM zinc for 24 h or with monoclonal anti-VEGF antibody (R&D Systems, Inc.) (kindly provided by F. Spinella, Regina Elena National Cancer Institute, Rome, Italy). Afterword, TNF-α was added for 48 h for DCs maturation and DC phenotype analyzed by flow cytometry. Phycoerytrin (PE)-conjugated anti-CD80 and FITC-conjugated anti-CD86 antibodies (Becton Dickinson, San Diego, CA) were used for cell surface staining. Cells were incubated with the antibodies for 30 minutes at 4°C and washed twice in PBS before analyses. DC were gated according to their FSC and SSC properties. Appropriate isotype controls were included and 5000 viable DC were acquired for each experiment. Flow cytometry acquisition was done with a cytofluorimeter EPICS XL Coulter (Hialeah, FL).

### Statistical Analysis

All experiment unless indicated were performed at least three times. All experimental results were expressed as the arithmetic mean and standard deviation (s.d.) of measurements was shown. Student’s *t*-test was used for statistical significance of the differences between treatment groups. Statistical analysis was performed using analysis of variance at 5% (p<0.05) or 1% (p<0.01).

**Figure 1:**
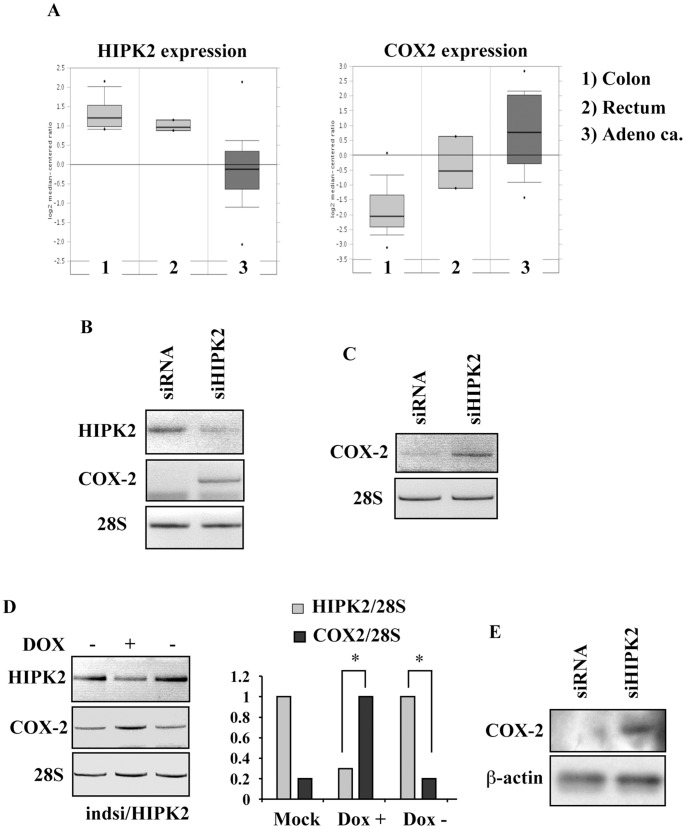
Inverse correlation between low HIPK2 and high COX-2 expression levels in cancer cells. (**A**) HIPK2 and COX-2 gene expression profile in primary tumors. The *box plots* represent HIPK2 (*left panel*) and COX-2 (*right panel*) mRNA levels in specimens of 1) colon, 2) rectum, and 3) colon adenocarcinoma primary tumors. *P<0.001. (**B**) Semi-quantitative RT-PCR analyses of HIPK2 and COX-2 gene expression in RKO cells stably interfered for HIPK2 function (siHIPK2) or with non-target RNA interference (siRNA). 28S was used as internal control. (**C**) Representative RT-PCR analysis of HCT116 cells transfected with pSuper-HIPK2 (siHIPK2) or pSuper control (siRNA) vector for 48 h. 28S was used as internal control. (**D, left panel**) Representative RT-PCR analyses of MCF7-indsi/HIPK2 cells treated with Dox (DOX+) for 5 days (for HIPK2 knockdown) and then cultured without Dox (DOX-) for additional 5 days (for HIPK2 recovery). (**D, right panel**) Densitometric analysis of HIPK2 and COX-2 levels (as in the left panel) was performed and normalized values to 28S mRNA levels were plotted. *P = 0.001. (**E**) Total cell extracts from RKOsiHIPK2 and control siRNA cells were analysed by Western immunoblotting to assess COX-2 protein levels. β-actin was used as protein loading control.

## Results and Discussion

### Inverse Correlation between Low HIPK2 and High COX-2 Expression Levels in Cancer Cells

To gain insights into the *in vivo* relationship between COX-2 and HIPK2 we performed an *in-silico* co-expression analysis comparing different microarray studies of various normal and tumor colon tissues from the Oncomine integrated cancer database research tool (http://www.oncomine.org). Analyses of datasets obtained from specimens of normal tissues and primary colon adenocarcinomas revealed an inverse correlation between HIPK2 and COX-2 expression. Thus, HIPK2 expression was high in normal tissues and significantly reduced in colon adenocarcinomas, while COX-2 expression was low in normal tissues and significantly increased in colon adenocarcinomas (**Fig. 1A**). We next attempted to investigate whether HIPK2 played a role in COX-2 regulation. As depicted in **Fig. 1B**, increased COX-2 expression levels were observed in RKO colon cancer cells stably interfered for HIPK2 function (siHIPK2) [29], compared to control siRNA cells. Similar inverse correlation between low HIPK2 and high COX-2 levels was found in HCT116 colon cancer cells undergoing transient HIPK2 depletion by siRNA (**Fig. 1C**), implying a role for HIPK2 in COX-2 mRNA expression in cancer cells. To further confirm this finding, we took advantage of the Dox-inducible MCF7 breast cancer cells (MCF7indsi/HIPK2), where Dox treatment induces HIPK2 downregulation [30], obtaining essentially similar results. Thus, Dox treatment (Dox +) reduced HIPK2 mRNA expression which significantly correlated with COX-2 upregulation, as also evidenced by the densitometric analysis (**Fig. 1D**). Subsequently, reversion of HIPK2 depletion by Dox removal (Dox -), restored the COX-2 gene expression levels similarly to the starting COX-2 levels of control cells, as also evidenced by densitometric analyses (**Fig. 1D**). Next, Western immunoblotting showed increased COX-2 protein levels in RKO HIPK2-depleted cells, compared to control siRNA cells (**Fig. 1E**). These results show, for the first time, an inverse correlation between HIPK2 and COX-2 expression in colon cancer cells which was intriguingly confirmed by probing the Oncomine dataset of normal and cancer tissues, indicating that this might be a typical cancer signature. Moreover, the effect of COX-2 upregulation in breast cancer cells following HIPK2 inhibition may be also taken in consideration.

**Figure 2:**
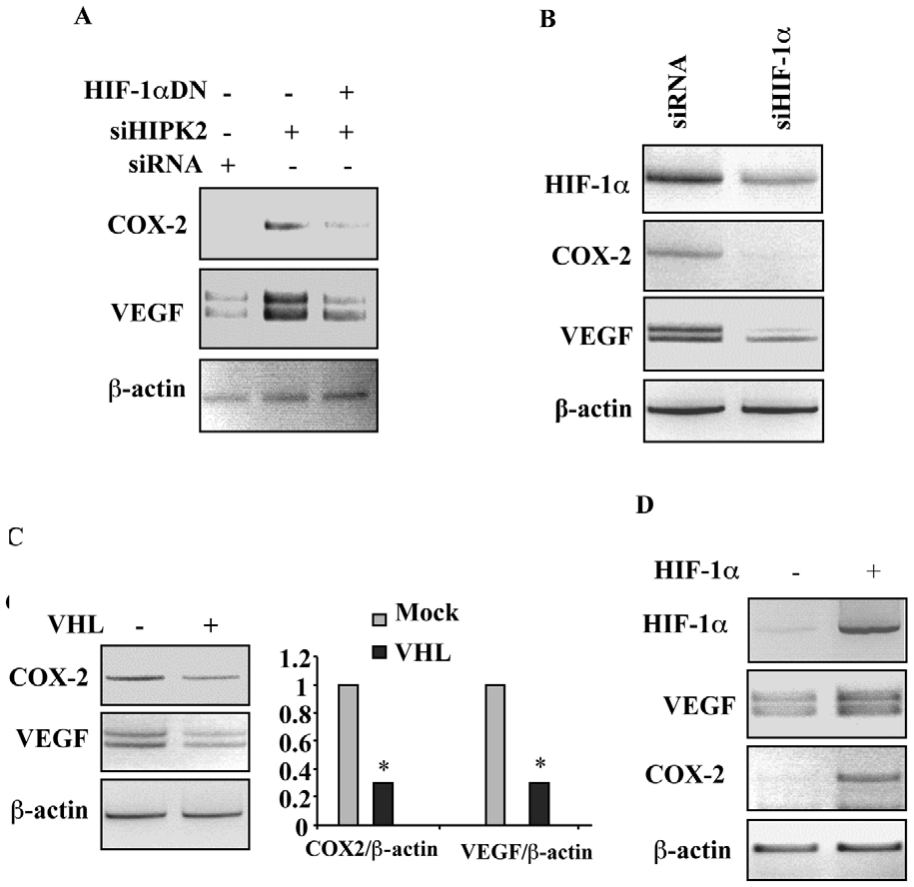
COX-2 upregulation in HIPK2 knockdown depends on HIF-1. (**A**) Representative RT-PCR analyses of control RKO-siRNA and RKO-siHIPK2 cells transfected with HIF-1α dominant negative (HIF-1αDN) for 36 h. The COX-2 and VEGF mRNA levels are shown. β-actin was used as internal control. (**B**) RT-PCR analyses of RKO-siHIPK2 cells were transfected with specific siRNA for HIF-1α knockdown or control siRNA. After transfection total RNA was extracted and RT-PCR was performed to assess HIF-1α, COX-2 and VEGF expression levels. β-actin was used as internal control. One representative experiment out of three independent experiments was shown. (**C, left panel**) RT-PCR analyses of RKO-siHIPK2 cells transfected with VHL expression vector (+) or with empty vector (-). After transfection total RNA was extracted and RT-PCR was performed to assess COX-2 and VEGF expression levels. β-actin was used as internal control. (**C, right panel**) Densitometric analysis of COX-2 and VEGF levels (as in the left panel) was performed and normalized values to β-actin mRNA levels were plotted. *P = 0.001. (**D**) 293 cells were transfected with HIF-1α expression vector (+) or with empty vector (−). After transfection total RNA was extracted and RT-PCR was performed to assess HIF-1α, COX-2 and VEGF expression levels. β-actin was used as internal control. One representative experiment out of three independent experiments was shown.

### Enhancement of COX-2 Expression in HIPK2 Knockdown Cells via HIF-1α

One of the mechanisms that enhance COX-2 transcription is through HIF-1 activity [20]. HIPK2 knockdown induces HIF-1 activity, seen as increased VEGF gene transcription and angiogenesis, by derepressing HIF-1α promoter transcription [6]. In order to examine if HIF-1α is involved in COX-2 expression, several genetic approaches were performed. RKO stably interfered for HIPK2 function (siHIPK2) were mainly used and transfection efficiency was monitored with the use of a co-transfected GFP vector throughout the experiments. To explore the effect of HIF-1α on the COX-2 gene transcription, we first carried out loss-of-function experiments with a dominant negative HIF-1α (HIF-1αDN) construct without DNA binding and transactivation domain that inhibits HIF-1 activity [32]. RT-PCR analysis showed that HIF-1αDN expression markedly reduced the levels of COX-2 in RKO-siHIPK2 cells (**Fig. 2A**), suggesting HIF-1 transcription-dependent COX-2 regulation. This effect was paralleled with the one observed on HIF-1 target gene VEGF (**Fig. 2A**). Then, HIF-1α depletion in RKO-siHIPK2 cells by specific siRNA interference showed that HIF-1α downregulation strongly inhibited both COX-2 and VEGF gene expression (**Fig. 2B**). As an additional approach to inhibit HIF-1 activity, we overexpressed von Hippel-Lindau (VHL) protein that has been shown to inhibit HIF-1 activity by targeting HIF-1α for proteasomal degradation [33]. As shown in **Fig. 2C** **,** VHL overexpression in RKO-siHIPK2 cells induced efficient COX-2 as well as VEGF downregulation, as evidenced by densitometric analyses. Finally, to further confirm the effect of HIF-1 in COX-2 regulation, we transfected HIF-1α expression vector in 293 cells observing that there was a significant enhancement of both COX-2 and VEGF mRNA expression, compared to control cells (**Fig. 2D**). Taken together, these results provide strong evidence that COX-2 upregulation, following HIPK2-knockdown, depended, al least in part, on HIF-1 activity as confirmed by the effect observed on HIF-1- target gene VEGF.

**Figure 3:**
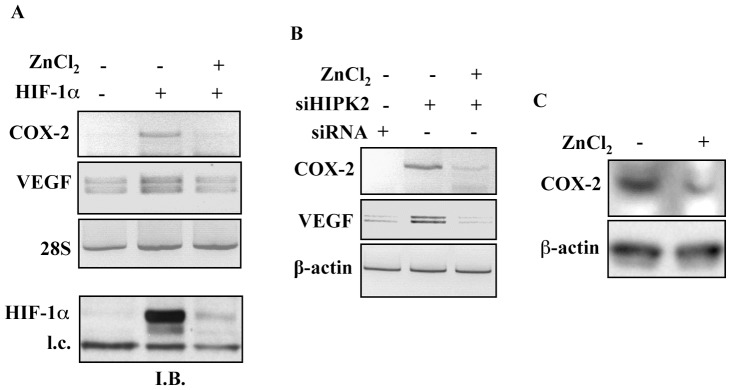
Zinc downregulates COX-2 expression levels in HIPK2 depleted cells. (**A**) 293 cells were transfected with HIF-1α expression vector (+) or with empty vector (−). HIF-1α-transfected cells were treated with ZnCl_2_ (100 µM). 24 h after treatment, cells were collected in divided in two for both RNA and protein analyses. RT-PCR was performed to assess COX-2 and VEGF expression levels (upper panel). 28S was used as internal control. Total cell extracts was analysed by Immunoblotting (I.B.) to assess HIF-1α protein levels. l.c. (loading control). (**B**) RKO-siHIPK2 cells were treated with ZnCl_2_ (100 µM) for 24 h. After transfection total RNA was extracted and RT-PCR was performed to assess COX-2 and VEGF expression levels. β-actin was used as internal control. One representative experiment out of three independent experiments was shown. (**C**) Total cell extracts from RKOsiHIPK2 untreated or treated with ZnCl_2_ (100 µM) for 24 h were analysed by Western immunoblotting to assess COX-2 protein levels. β-actin was used as protein loading control.

### Zinc Downregulates COX-2 Expression Levels in HIPK2 Depleted Cells

We previously demonstrated that zinc ions supplementation in constitutively hypoxic prostate cancer cells or in angiogenic glioblastoma cells downregulates HIF-1α expression, consequently inhibiting HIF-1 activity [8]. This finding was next substantiated by a genome-wide analyses where zinc indeed counteracts the gene expression profiles induced by hypoxia-activated HIF-1 [34]. Here we first overexpressed HIF-1α in 293 cells and analyzed mRNA levels by semiquantitative RT-PCR before and after zinc treatment. As shown in **Fig. 3A**, HIF-1-induced COX-2 and VEGF gene expression was efficiently inhibited by zinc treatment. The HIF-1α overexpression and its downregulation upon zinc treatments, as previously reported [8], were shown by Western immunoblotting (I.B.) (**Fig. 3A** **, lower panel**). Then, zinc treatment of RKO-siHIPK2 cells showed significant reduction of both COX-2 and VEGF mRNA gene expression by RT-PCR analysis (**Fig. 3B**). Consequently, Western immunoblotting confirmed COX-2 downregulation in HIPK2 depleted cells after zinc treatment (**Fig. 3C**). Of note, in our hands zinc treatment (100 µM for 24 h) did not affect cell viability (data not shown). Because COX-2 is often upregulated in tumors leading to tumor aggressiveness and bad prognosis [14], targeting COX-2 may be an important achievement in cancer therapy. Thus, COX-2 selective inhibitors have shown potent antitumor effects in several different animal models of colorectal cancer; in agreement, deletion of the COX-2 gene results in a marked decrease of adenoma burden in both small and large intestine in *Apc^Δ716^* mutant mice and in *Apc^min^* mice [35]. Nevertheless selective COX-2 inhibitors were shown to be highly effective in preventing polyp recurrence, the associated adverse cardiovascular side effects by these drugs may affect appropriate randomised clinical trials [36], [37]. Therefore, it is mandatory to develop new approaches to target COX-2 function. In this regard, the use of zinc in downregulating HIF-1-induced COX-2 expression may represent an interesting starting-point to overcome the adverse effects of at least some specific COX-2 inhibitors; moreover by acting on HIF-1 activity zinc may affect several interconnected signaling pathways. Finally, whether zinc might inhibit COX-2 also independently from HIF-1 needs to be further elucidated.

**Figure 4:**
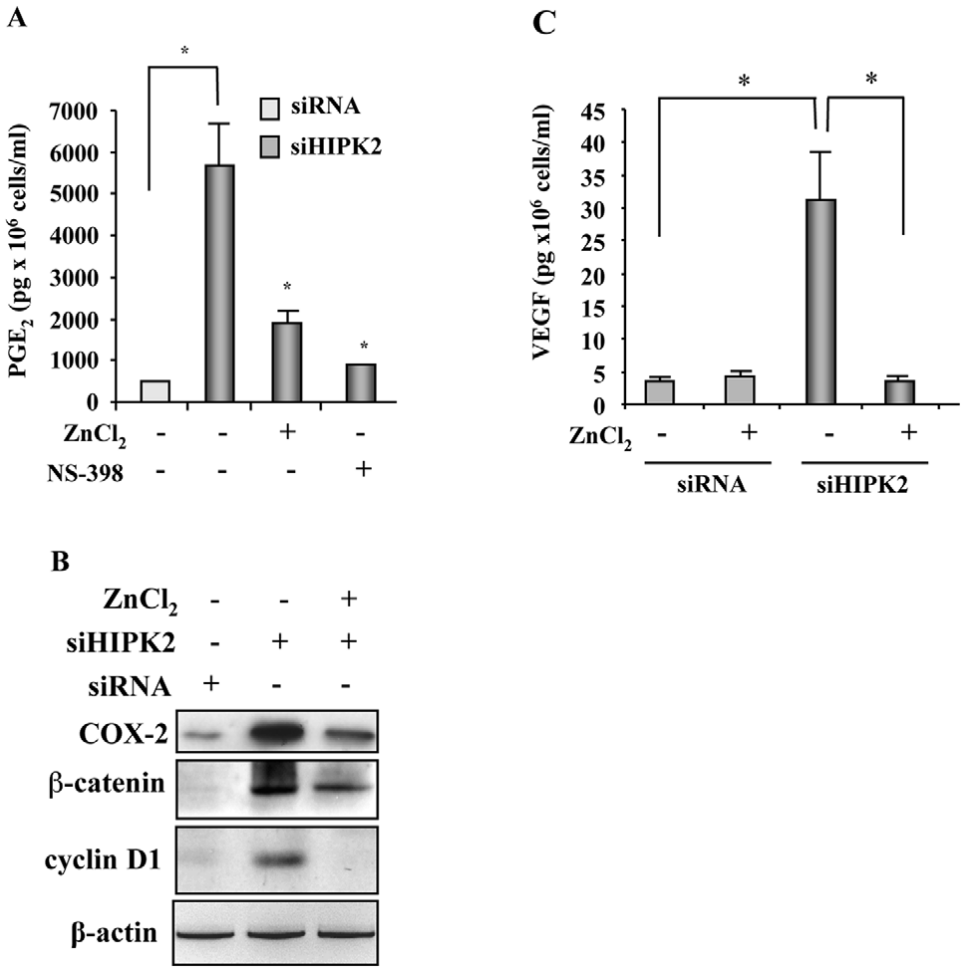
Zinc inhibits PGE_2_ signaling pathways in HIPK2 depleted cells. (**A**) PGE_2_ production was assayed by ELISA in RKO-siRNA control cells left untreated and in HIPK2 depleted cells (siHIPK2) before and after ZnCl_2_ treatment (100 µM for 24 h) and after treatment with COX-2 specific inhibitor NS-398 (50 nM) for 24 h. PGE_2_ production was plotted as pg/10^6^ cells. Columns, mean of two independent experiments performed in duplicate, ±S.D. *P<0.001. (**B**) Total cell extracts from RKO-siRNA control cells left untreated and HIPK2 depleted cells (siHIPK2) before and after ZnCl_2_ treatment (100 µM for 24 h) were analysed by Western immunoblotting to assess COX-2, β-catenin, and cyclin D1 protein levels. β-actin was used as protein loading control. (**C**) VEGF production was assayed by ELISA in RKO-siRNA control cells and in HIPK2 depleted cells (siHIPK2) before and after ZnCl_2_ treatment (100 µM for 24 h). VEGF production was plotted as pg/10^6^ cells. Columns, mean of two independent experiments performed in duplicate, ±S.D. *P<0.001.

### Zinc Inhibits PGE_2_ Signaling Pathways in HIPK2-depleted Cells

Having established that zinc may downregulate COX-2 levels in HIPK2 depleted cells, we next sought to evaluate PGE_2_ modulation. ELISA assay showed that the relevant amount of PGE_2_ produced by HIPK2 depleted compared to control cells, as previously reported [10], was significantly inhibited by zinc supplementation (**Fig. 4A**). Interestingly, zinc-induced PGE_2_ inhibition was as efficient as the specific COX-2 inhibitor NS-398 (**Fig. 4A**). In agreement, western immunoblotting showed strong reduction of COX-2 protein levels after zinc treatment (**Fig. 4B**), as reported above. Moreover, the enhanced levels of β-catenin and of its target cyclin D1 in HIPK2 depleted cells, as previously reported [3], [4], were markedly inhibited by zinc treatment (**Fig. 4B**), which paralleled the above PGE_2_ inhibition. This is an interesting correlation, because PGE_2_ has been shown to activate β-catenin as transcription factor to induce the expression of several genes, including COX-2 and cyclin D1, generating a positive autoregulatory loop which favours tumour progression [12], [38].

**Figure 5:**
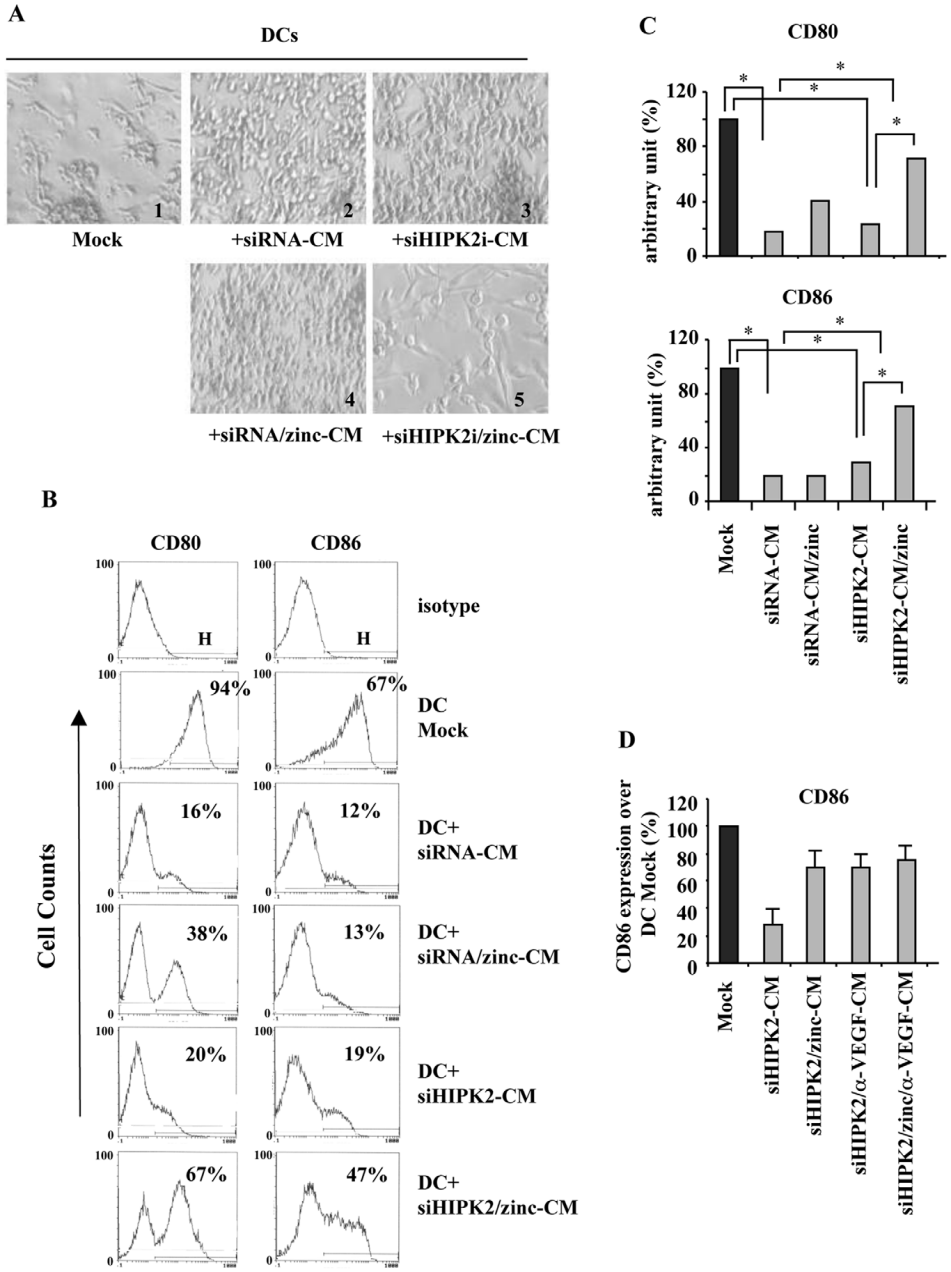
Inhibition/reactivation of DCs maturation cultured with conditioned media (CM) of siRNA control or HIPK2 depleted cells with or withour zinc treatment. (**A**) Light microscopy showing control differentiated DCs (Mock, panel 1) and inhibition of differentiated DCs morphology in the presence of CM of siRNA control (panel 2) or HIPK2 depleted cells (siHIPK2) (panel 3); this inhibition did not take place only if DCs where cultured in the presence of CM of zinc-treated siHIPK2-CM (panel 5) compared to CM of zinc-treated siRNA control cells (panel 4). One representative experiment out of three independent experiments was shown. Microphotograph magnification, 10×. (**B**) Analysis of surface DCs maturation markers, CD80 and CD86, using flow cytometry. DC Mock: control mature DCs; DC+siRNA-CM: DCs maturation in the presence of CM of siRNA control cells; DC+siHIPK2-CM: DCs maturation in the presence of CM of HIPK2 depleted cells (siHIPK2); DC+siRNA/zinc-CM: DCs maturation in the presence of zinc-treated siRNA cells; DC+siHIPK2/zinc-CM: DCs maturation in the presence of CM of zinc-treated siHIPK2 cells. A representative experiment, out of three, is shown. The isotype control is shown. H indicates the region used to calculate the % of positive cells for each costimulatory molecule, for each sample. (**C**) Percentage of CD80 and CD86 expression in DCs cultured with the indicated CM as in (B) and compared to control mature DCs fixed at 100%. (**D**) Analysis of surface DCs maturation marker CD86 using flow cytometry. The results are expressed as percentage (%) ±S.D., over DC Mock fixed at 100%. Mock: control mature DCs; siHIPK2-CM: DCs maturation in the presence of CM of HIPK2 depleted cells (siHIPK2); siHIPK2/zinc-CM: DCs maturation in the presence of CM of zinc-treated siHIPK2 cells; siHIPK2/α-VEGF-CM: DCs maturation in the presence of CM of α-VEGF-treated HIPK2 depleted cells; siHIPK2/zinc/α-VEGF-CM: DCs maturation in the presence of CM of zinc- and α-VEGF-treated HIPK2 depleted cells.

COX-2/PGE_2_ axis has been shown to induce VEGF and promote angiogenesis [13]. Therefore, VEGF production was analysed in HIPK2 depleted cells. ELISA assay showed that the high levels of VEGF produced in HIPK2 depleted cells, compared to control cells, were significantly inhibited by zinc supplementation (**Fig. 4C**). Taken together, these results provide evidence that the signaling pathways activated in our model of constitutive hypoxic HIPK2 depleted cells, such as PGE_2_ and VEGF, may be inhibited by zinc supplementation, likely by acting on HIF-1 activity. These findings thus strengthen the role of HIPK2 in curbing tumor progression [39]. However, given that HIPK2 is one of the many factors related to COX-2/PGE_2_ pathway activation in cancer, it will be interesting in future studies to evaluate the effect of zinc treatment in cancer cells with upregulated COX-2/PGE_2_ pathway independently from HIPK2.

**Figure 6:**
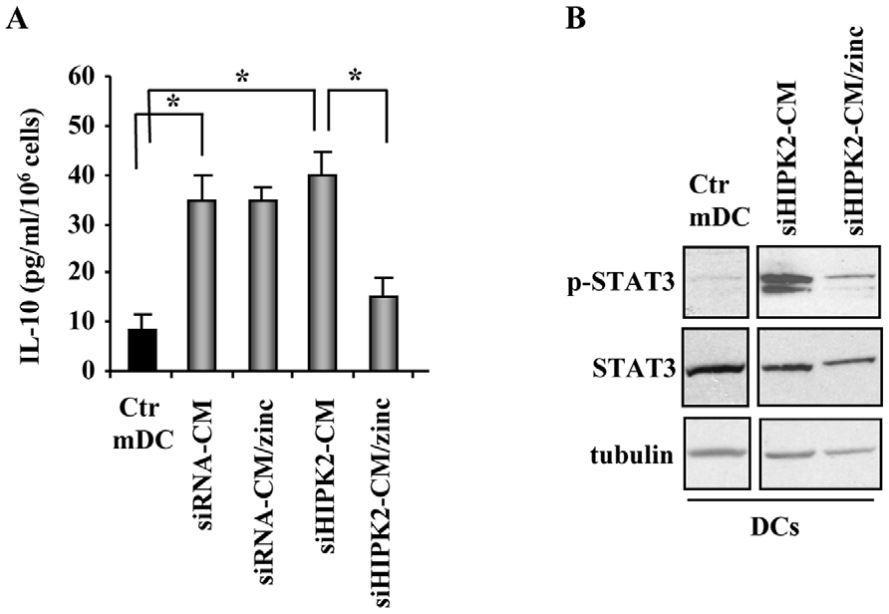
Molecular mechanisms of DCs inhibition/reactivation. (**A**) IL-10 production by mature DCs cultured with CM of siRNA or siHIPK2 cells (plus or minus zinc-treatment), assayed by ELISA. Columns are mean of three independent experiments performed in duplicate. *P <0.001. (**B**) Western blot analysis of p-STAT3 and total STAT3 in control (Ctr) mature DCs, or DCs cultured with CM of siHIPK2 cells with or without zinc-treatment. Tubulin was used as protein loading control.

### Zinc Reduces the Ability of HIPK2 Depleted Cells to Suppress DCs Maturation

We next wanted to test whether conditioned media (CM) of zinc-treated or -untreated cells, as showed in the above paragraph, could affect immune cells and in particular DCs maturation. It is well established that tumor-host interaction may lead to immunosuppression [23]. In particular, tumors synthesize and secrete factors that may suppress DCs function to prevent immune response [27], [28]. To mimic an *in vivo* condition, we decided to assess the differentiation of DCs in the presence of 20% CM of siHIPK2 or si-RNA control cells cultured with or without zinc. To this aim, the CM showed in **Fig. 4A** and **Fig. 4C** were used to take advantage of the zinc-induced modulation of COX-2/PGE_2_ and VEGF pathways. Thus, inflammatory mediators in the tumour microenvironment other than promoting tumour growth and vascular development have been shown to enable evasion of anti-tumour immune responses [23], [26]. Interestingly, we first noticed a strong inhibition of differentiated DCs morphology when DCs maturation was allowed in the presence of CM of both HIPK2-depleted and siRNA control cells (**Fig. 5A** **, compare panel 1 with panel 2 and 3**). However, this inhibition did not take place if DCs were cultured in the presence of CM of zinc-treated siHIPK2 cells (**Fig. 5A** **, compare panel 3 with panel 5**), while inhibition of differentiated DCs morphology did remain when DCs were cultured in the presence of CM of zinc-treated siRNA control cells (**Fig. 5A** **, compare panel 2 with panel 4**). These observations are in line with the concept that tumors produce immunosuppressive factors that may inhibit DCs maturation [26]–[28], [40]. Moreover, they also suggest that the immunosuppressive pathways induced by HIPK2 knockdown rather than those produced by siRNA control cells could be preferentially targeted by zinc for DCs reactivation. Therefore, the different outcome in DCs maturation achieved in the presence of supernatants of zinc-treated control or HIPK2 depleted cells established the specificity of zinc action, rather than a direct DCs activation by zinc which, after 4 days colture, might still remain in conditioned media from cancer cells. In particular, the zinc-targeted PGE_2_ and VEGF pathways, produced after HIPK2 depletion (**Fig. 4**), could have a role in DCs maturation, although their direct role and/or the presence and modulation of additional immunosuppressive factors produced in our experimental model need to be elucidated.

We then determined the phenotype of DCs differentiation by assessing the surface expression of co-stimulatory CD80 and CD86 molecules using flow cytometry analyses. As shown in **Fig. 5B** **and** **Fig. 5C**, CD80 and CD86 expression was significantly inhibited when DCs maturation was allowed in the presence of CM of either siRNA (siRNA-CM) or siHIPK2 (siHIPK2-CM) cells, compared to control mature DCs (DC Mock). In agreement with our hypothesis, CD80 and CD86 expression significantly increased when DCs maturation was allowed in the presence of CM of zinc-treated siHIPK2 cells, while CD80 and CD86 expression did not essentially change when DCs maturation was allowed in the presence of CM of zinc-treated siRNA control cells (**Fig. 5B** **and** **Fig. 5C**). CD80 and CD86 expression, when DCs maturation was allowed in the presence of CM of zinc-treated siHIPK2 cells, reached almost 70% of that of the control mature DCs. These findings indicate that zinc could inhibit some immunosuppressive pathways produced by HIPK2-depleted cells to reactivate DCs maturation. As mentioned above, this effect might depend by zinc-induced inhibition of immunosuppressive molecules such as PGE_2_ and VEGF (**Fig. 4**). Thus, PGE_2_ has been shown to induce DCs dysfunction [24], [25]. Similarly, VEGF has been shown to inhibit the functional maturation of DCs [41]. In this regard, VEGF inhibition in HIPK2-depleted cell culture, by the use of anti-VEGF monoclonal antibody, efficiently restored DCs maturation, seen as expression of CD86, in a similar extent to that obtained by CM of zinc-treated siHIPK2 cells (**Fig. 5D**). Similar results were obtained for CD80 expression (data not shown). However, the role of additional immunosuppressive pathways produced by HIPK2 knockdown cannot be excluded and needs to be further elucidated.

In the attempt to evaluate the molecular mechanism involved in reactivation of DCs maturation, we first assessed IL-10 production by DCs challenged with the conditioned media of siRNA control or siHIPK2 depleted cells with or without zinc treatment, as endogenous IL-10 appears to be and important regulator of DCs biology [42]. ELISA assay showed that immunosuppressive cytokine IL-10 was similarly produced when DCs maturation was allowed in the presence of CM of either siRNA control or siHIPK2 cells (**Fig. 6A**). Interestingly, the release of IL-10 by DCs was significantly inhibited only when DCs maturation was allowed in the presence of CM of zinc-treated siHIPK2 cells, while CM of zinc-treated siRNA control cells did not have such an effect (**Fig. 6A**). As inflammatory molecules such as IL-6, IL-10, and VEGF may activate STAT3 whose abnormal phosphorylation/activation is reported to be the underlying mechanism involved in impairment of DCs maturation [43], [44], STAT3 phosphorylation (p-STAT3-Y705) was analysed in mature control DCs compared to DCs cultures with CM of siHIPK2 depleted cells treated or not with zinc. As shown in **Fig. 6B**, strong STAT3 phosphorylation was evidenced when DCs were cultured with CM of siHIPK2 cells, compared to the control mature DCs (**Fig. 6B**). Strong inhibition of STAT3 phosphorylation was obtained when DCs were cultured with CM of zinc-treated siHIPK2 depleted cells (**Fig. 6B**). Therefore, zinc-induced modulation of PGE_2_ and VEGF production (see **Fig. 4**) might correlate with DCs dysfunction. Many tumor-produced factors, such as IL-10, IL-6 and VEGF, which are crucial for both tumour growth and immunosuppression, activate STAT3 to create an efficient ‘feedforward’ mechanism to ensure increased STAT3 activity both in tumour cells and in tumour-associated immune cells which, in turn, generates immunosuppression involving both innate and adaptive immunity [44]. Thus, hyperactivation of STAT3 is involved in abnormal differentiation of dendritic cells in cancer [45]. In this regard, we recently found that release of cytokines such as IL-6, IL-10 and VEGF by Primary Effusion Lymphoma (PEL) cells suppresses DCs differentiation by activating STAT3 and p38 MAPK molecules [46], [47]. Therefore, STAT3 inhibitors, other than acting as a chemotherapeutic drug, can improve the DCs activity against some type of tumors [44], [48].

In conclusion, in this study we found that HIPK2 knockdown induced COX-2 upregulation, mostly depending on HIF-1 activity. This finding supports the PGE_2_ production after HIPK2 depletion, as previously reported [10]. Recent studies demonstrate that HIPK2 inhibition does exist in human tumors and depends by several mechanisms including HIPK2 cytoplasmic localization, protein degradation [2], and loss of heterozygosity (LOH) [49], recapitulating the biological outcome obtained by RNA interference studies in tumor cells, such as p53 inactivation, resistance to therapies, apoptosis inhibition, and tumor progression [2]. The role of HIPK2 in modifying molecular pathways to restrain tumor development is also confirmed by studies with HIPK2 knockout mice [50], [51]. Therefore, HIPK2 inhibition becomes an additional factor that might be related to COX-2/PGE_2_ pathways activation *in vivo*. This finding was corroborated in this study by analyses of Oncomine dataset of normal and cancer tissues (**Fig. 1A**), indicating that low HIPK2 expression and high COX-2 expression might be a typical cancer signature. This finding also strengthens the role of HIPK2 as oncosuppressor and as a molecule to be exploited and/or (re)activated for cancer therapy [52]. Moreover, the present study suggests that HIPK2 inhibition might be a possible mechanism of immune deregulation which will be worth to further evaluate in human studies. However, while regulation of HIPK2 expression is still elusive, HIPK2 protein degradation has been demonstrated under hypoxia [39], [53] which is a condition often present in solid tumors [5], [22]. Hypoxia within the tumor microenvironment is correlated with poor treatment outcome, with activation or inactivation of molecular pathways involved in tumor progression, but also with immunosuppression including impaired dendritic cell maturation [54]. Therefore, targeting hypoxia-induced HIF-1 is a functional strategy to abolish interconnected pathways involved in tumor progression, such as COX-2/PGE_2_/VEGF, which in turn may lead to immunosuppression.

Inflammatory mediators have been shown to hold the key to dendritic cell suppression and tumor progression [26]. Zinc has been shown to have an anti-inflammatory role [55], [56], however, further studies in different cancer cells are needed to corroborate our working model of zinc inhibition of tumor-induced pro-inflammatory pathways for efficient DCs activation and more interestingly for the induction of immunogenic cell death, as recently demonstrated by our studies in PEL with Bortezomib and STAT3 inhibitor [57], for efficient tumor regression.

## References

[1] Puca R, Nardinocchi L, Sacchi A, Rechavi G, Givol D, et al (2009) HIPK2 modulates p53 activity towards pro-apoptotic transcription. Mol Cancer 14 8: 85. 10.1186/1476-4598-8-85 PMC2768676 19828042

[2] Puca R, Nardinocchi L, Givol D, D’Orazi G (2010) Regulation of p53 by HIPK2: molecular mechanisms and therapeutical implications in human cancer cells. Oncogene 29: 4378–4387. 20514025 10.1038/onc.2010.183

[3] Puca R, Nardinocchi L, D’Orazi G (2008) Regulation of vascular endothelial growth factor expression by homeodomain-interacting protein kinase 2. J Exp Clin Cancer Res 27: 1–6. 10.1186/1756-9966-27-22 PMC2494538 18644116

[4] Kim EA, Kim JE, Sung KS, Choi DW, Lee BJ, et al (2010) Homeodomain-interacting protein kinase 2 (HIPK2) targets β-catenin for phosphorylation and proteasomal degradation. Biochem Biophys Res Commun 394: 966–971. 20307497 10.1016/j.bbrc.2010.03.099

[5] Semenza GL (2010) Defining the role of hypoxia-inducible factor 1 in cancer biology and therapeutics. Oncogene 29: 625–634. 19946328 10.1038/onc.2009.441 PMC2969168

[6] Nardinocchi L, Puca R, Guidolin D, Belloni AS, Bossi G, et al (2009) Transcriptional regulation of hypoxia-inducible factor 1alpha by HIPK2 suggests a novel mechanism to restrain tumor growth. Biochem Biophys Acta Molecular Cell Research 1793: 368–377. 10.1016/j.bbamcr.2008.10.013 19046997

[7] Nardinocchi L, Puca R, Sacchi A, D’Orazi G (2009) Inhibition of HIF-1alpha activity by homeodomain-interacting protien kinase-2 correlates with sensitization of chemoresistant cells to undergo apoptosis. Mol Cancer 8: 1–8. 19128456 10.1186/1476-4598-8-1 PMC2628864

[8] Nardinocchi L, Pantisano V, Puca R, Porru M, Aiello A, et al. (2010) Zinc downregulates HIF-1alpha and inhibits its activity in tumor cells in vitro and in vivo. PLoS ONE 5(12)e15048. 10.1371/journal.pone.0015048 PMC3001454 21179202

[9] Nardinocchi L, Puca R, Sacchi A, Rechavi G, Givol D, et al (2009) Targeting hypoxia in cancer cells by restoring homeodomain-interacting protein kinase-2 and p53 activity and suppressing HIF-1alpha. PloS ONE 4(8): e6819. 19714248 10.1371/journal.pone.0006819 PMC2729407

[10] D’Orazi G, Sciulli MG, Di Stefano V, Riccioni S, Frattini M, et al (2006) Homeodomain-interacting protein kinase-2 restrains cytosolic phospholipase A2-dependent prostaglandin E2 generation in human colorectal cancer cells. Clin Cancer Res 12: 735–741. 16467083 10.1158/1078-0432.CCR-05-1557

[11] Wang D, DuBois RN (2010) Eicosanoides and cancer. Nat Rev Cancer 10: 181–193. 20168319 10.1038/nrc2809 PMC2898136

[12] Chan TA (2006) Prostaglandin and the colon cancer connection. TRENDS Mol Med 12: 240–244. 16650804 10.1016/j.molmed.2006.04.006

[13] Wang D, DuBois RN (2004) Cyclooxygenase 2-derived prostaglandin E2 regulates the angiogenic switch. Proc Natl Acad Sci USA 101: 415–416. 14707264 10.1073/pnas.0307640100 PMC327160

[14] DuBois RN, Abramson SB, Crofford L, Gupta RA, Simon LS, et al (1998) Cyclooxygenase in biology and diseases. FASEB J 12: 1063–1073. 9737710

[15] Eberhart CE, Coffey RJ, Radhika A, Giardiello FM, Ferrenbach S, et al (1994) Up-regulation of cyclooxygenase-2 gene expression in human colorectal adenomas and adenocarcinomas. Gastroenterology 107: 1183–1188. 7926468 10.1016/0016-5085(94)90246-1

[16] Ristimaki A, Honkanen N, Jankala H, Sipponen P, Harkonen M (1997) Expression of cyclooxygenase-2 in human gastric carcinoma. Cancer Res 57: 1276–1280. 9102213

[17] Saukkonen K, Rintahaka J, Sivula A, Buskens CJ, Van Rees BP, et al (2003) Cyclooxygenase-2 and gastric carcinogenesis. APMIS 111: 915–925. 14616542 10.1034/j.1600-0463.2003.1111001.x

[18] Smith WL, DeWitt DL, Garavito RM (2000) Cyclooxygenases: structural, cellular, and molecular biology. Annu Rev Biochem 69: 145–182. 10966456 10.1146/annurev.biochem.69.1.145

[19] Nuñez F, Bravo S, Cruzat F, Montecino M, De Ferrari GV (2011) Wnt/β-catenin signaling enhances cyclooxygenase-2 (COX2) transcriptional activity in gastric cancer cells. PLoS ONE 6(4): e18562. 21494638 10.1371/journal.pone.0018562 PMC3071840

[20] Kaidi A, Qualtrough D, Williams AC, Paraskeva C (2006) Direct transcriptional up-regulation of cyclooxygenase-2 by hypoxia-inducible factor (HIF)-1 promotes colorectal tumor cell survival and enhances HIF-1 transcriptional activity during hypoxia. Cancer Res 66: 6683–6691. 16818642 10.1158/0008-5472.CAN-06-0425

[21] Tsujii M, Kawano S, Tsuji S, Sawaoka H, Hori M, et al (1998) Cyclooxygenase regulates angiogenesis induced by colon cancer cells. Cell 93: 705–716. 9630216 10.1016/s0092-8674(00)81433-6

[22] Semenza GL (2003) Targeting HIF-1 for cancer therapy. Nat Rev Cancer 94: 1021–1028. 10.1038/nrc1187 13130303

[23] Hanahan D, Weinberg RA (2011) Hallmarks of cancer: the next generation. Cell 144: 646–674. 21376230 10.1016/j.cell.2011.02.013

[24] Greenhough A, Smartt HJM, Moore AE, Roberts HR, Williams AC, et al (2009) The COX-2/PGE2 pathway: key roles in the hallmarks of cancer and adaptation to the tumor microenvironment. Carcinogenesis 30: 377–386. 19136477 10.1093/carcin/bgp014

[25] Kalinski P (2012) Regulation of immune response by prostaglandin E2. J Immunol 188: 21–28. 22187483 10.4049/jimmunol.1101029 PMC3249979

[26] Sheng KC, Wright MD, Apostolopoulos V (2011) Inflammatory mediators hold the key to dendritic cell suppression and tumor progression. Curr Med Chem 18: 5507–5518. 22172061 10.2174/092986711798347207

[27] Gabrilovich D (2004) Mechanisms and functional significance of tumor-induced dendritic-cell defects. Nat Rev Immunol 4: 941–952. 15573129 10.1038/nri1498

[28] Gottfried E, Kreutz M, Mackensen A (2008) Tumor-induced modulation of dendritic cell function. Cytokine &Growth Fact Rev 19: 65–77. 10.1016/j.cytogfr.2007.10.008 18061513

[29] Di Stefano V, Rinaldo C, Sacchi A, Soddu S, D’Orazi G (2004) Homeodomain-interacting protein kinase-2 activity and p53 phosphorylation are critical events for cisplatin-mediated apoptosis. Exp Cell Res 293: 311–320. 14729469 10.1016/j.yexcr.2003.09.032

[30] Puca R, Nardinocchi L, Bossi G, Rechavi G, Givol D, et al (2009) Restoring wtp53 activity in HIPK2 depleted MCF7 cells by modulating metallothionein and zinc. Exp Cell Res 315: 67–75. 18996371 10.1016/j.yexcr.2008.10.018

[31] Chen C, Okayama H (1987) High-efficiency transformation of mammalian cells by plasmid DNA. Mol Cell Biol 7: 2745–2752. 3670292 10.1128/mcb.7.8.2745-2752.1987 PMC367891

[32] Zhong XS, Liu LZ, Skinner HD, Cao Z, Ding M, et al (2007) Mechanism of vascular endothelial growth factor expression mediated by cisplatin in human ovarian cancer cells. Biochem Biophys Res Commun 358: 92–98. 17470361 10.1016/j.bbrc.2007.04.083

[33] Maxwell PH, Wiesener MS, Chang GW, Clifford SC, Vaux EC, et al (1999) The tumour suppressor protein VHL targets hypoxia-inducible factors for oxygen-dependent proteolysis. Nature 399: 71–75. 10.1038/20459 10353251

[34] Sheffer M, Simon AJ, Rechavi G, Domany E, Givol D, et al (2011) Genome-wide analysis discloses reversal of the hypoxia-induced changes of gene expression in colon cancer cells by zinc supplementation. Oncotarget 2: 1191–1202. 22202117 10.18632/oncotarget.395 PMC3282077

[35] Oshima M, Dinchuk JE, Kargman SL, Oshima H, Hancock B, et al (1996) Suppression of intestinal polyposis in *Apc^Δ716^* knockout mice by inhibition of cyclooxygenase 2 (COX-2). Cell 87: 803–809. 8945508 10.1016/s0092-8674(00)81988-1

[36] Garcia Rodriguez LA, Patrignani P (2006) The ever growing story of cyclo-oxygenase inhibition. The Lancet 368: 1745–1747. 10.1016/S0140-6736(06)69667-0 17113403

[37] Cha DYI, DuBois RN (2007) NSAID and cancer prevention: targets downstream of COX-2. Annu Rev Med 58: 239–252. 17100552 10.1146/annurev.med.57.121304.131253

[38] Castellone MD, Teramoto H, Williams BO, Druey KM, Gutkind JS (2005) Prostaglandin E2 regulates colon cancer cell growth through a Gs-axin-β-catenin signalling axis. Science 310: 1504–1510. 16293724 10.1126/science.1116221

[39] Nardinocchi L, Puca R, Givol D, D’Orazi G (2010) HIPK2-a therapeutical target to be (re)activated for tumor suppression: role in p53 activation and HIF-1α inhibition. Cell Cycle 9: 1270–1275. 20234185 10.4161/cc.9.7.11125

[40] Rabinovich GA, Gabrilovich D, Sotomayor EM (2007) Immunosuppressive strategies that are mediated by tumor cells. Annu Rev Immunol 25: 267–296. 17134371 10.1146/annurev.immunol.25.022106.141609 PMC2895922

[41] Gabrilovich DI, Chen HL, Girgis KR, Cunningham HT, Meny GM, et al (1996) Production of vascular endothelial growth factor by human tumors inhibits the functional maturation of dendritic cells. Nat Med 2: 1096–1103. 8837607 10.1038/nm1096-1096

[42] Corinti S, Albanesi C, la Sala A, Pastore S, Girolomoni G (2001) Regulatory activity of autocrine IL-10 on dendritic cell functions. J Immunol 166: 4312–4318. 11254683 10.4049/jimmunol.166.7.4312

[43] Wang T, Niu G, Kortylewski M, Burdelya L, Shain K, et al (2004) Regulation of the innate and adaptive immune responses by Stat-3 signaling in tumor cells. Nat Med 10: 48–54. 14702634 10.1038/nm976

[44] Yu H, Kortylewski M, Pardoll D (2007) Crosstalk between cancer and immune cells: role of STAT3 in the tumour microenvironment. Nat Rev Immunol 7: 41–51. 17186030 10.1038/nri1995

[45] Nefedova Y, Huang M, Kusmartsev S, Battacharya R, Cheng P, et al (2004) Hyperactivation of STAT3 is involved in abnormal differentiation of dendritic cells in cancer. J Immunol 172: 464–474. 14688356 10.4049/jimmunol.172.1.464

[46] Cirone M, Lucania G, Aleandri S, Borgia G, Trivedi P, et al (2008) Suppression of dendritic cell differentiation through cytokines released by Primary Effusion Lymphoma cells. Immunol Lett 120: 37–41. 18680763 10.1016/j.imlet.2008.06.011

[47] Cirone M, Di Renzo L, Trivedi P, Lucania G, Borgia G, et al (2010) Dendritic cell differentiation blocked by primary effusion lymphoma-released factors is partially restored by inhibition of p38 MAPK. Int J Immunopathol Pharmacol 23: 1079–1086. 21244757 10.1177/039463201002300412

[48] Nefedova Y, Cheng P, Gilkes D, Blaskovich M, Beg AA, et al (2005) Activation of dendritic cells via inhibition of Jak2/STAT3 signaling. J Immunol175: 4338–4364. 10.4049/jimmunol.175.7.4338 PMC1351251 16177074

[49] Lavra L, Rinaldo C, Ulivieri A, Luciani E, Fidanza P, et al (2011) The loss of the p53 activator HIPK2 is responsible for galectin-3 overexpression in well differentiated thyroid carcinomas. PLoS ONE 6(6): e20665. 21698151 10.1371/journal.pone.0020665 PMC3117790

[50] Wei G, Ku S, Ma GK, Saito S, Tang AA, et al (2007) HIPK2 represses β-catenin-mediated transcription, epidermal stem cell expansion, and skin tumorigenesis. Proc Natl Acad Sci USA 104: 13040–13045. 17666529 10.1073/pnas.0703213104 PMC1936219

[51] Mao JH, Wu D, Kim IJ, Kang HC, Wei G, et al (2011) Hipk2 cooperates with p53 to suppress γ-ray radiation-induced mouse thymic lymphoma. Oncogene 31: 1176–1180. 21785465 10.1038/onc.2011.306 PMC3307058

[52] D’Orazi G, Rinaldo C, Soddu S (2012) Updates on HIPK2: a resourceful suppressor for clearing cancer. J Exp Clin Cancer Res 31: 63. 22889244 10.1186/1756-9966-31-63 PMC3432601

[53] Calzado MA, de la Vega L, Moller A, Bowtell DD, Schmitz ML (2009) An inducible autoregulatory loop between HIPK2 and Siah2 at the apex of the hypoxic response. Nat Cell Biol 11: 85–91. 19043406 10.1038/ncb1816

[54] Lee CT, Mace T, Repasky EA (2010) Hypoxia-driven immunosuppression: A new reason to use thermal therapy in the treatment of cancer? Int J Hyperthermia 26: 232–246. 20388021 10.3109/02656731003601745 PMC2909041

[55] Prasad A (2008) Clinical, immunological, anti-inflammatory and antioxidant roles of zinc. Exp Gerontol 43: 370–3. 18054190 10.1016/j.exger.2007.10.013

[56] Fong LYY, Zhang L, Jiang Y, Farber JL (2005) Dietary zinc modulation of COX-2 expression and lingual and esophageal carcinogenesis in rats. J Natl Cancer Inst 97: 40–50. 15632379 10.1093/jnci/dji006

[57] Cirone M, Di Renzo L, Lotti LV, Conte V, Trivedi P, et al (2012) Primary effusion Lymphoma cell death induced by Bortezomib and AG 490 activates dendritic cells through CD91. PLoS ONE 7(3): e31732. 22412839 10.1371/journal.pone.0031732 PMC3296697

